# Novel Insights into Pediatric Acute Lymphoblastic Leukemia Ophthalmic Relapses from a Nationwide Cohort Study

**DOI:** 10.7150/jca.64996

**Published:** 2022-01-24

**Authors:** Solenne Le Louet, Véronique Icart, Marion Strullu, Arnaud Petit, Claire Freycon, Pascale Blouin, Jill Serre, Nicolas Rama, Yves Reguerre, Christophe Piguet, Marlène Pasquet, Audrey David, Pauline Simon, Marilyne Poiree, Liana Carausu, Fanny Rialland, Wadih Abouchahla, Paul Saultier, Stéphane Ducassou, Julie Valduga, André Baruchel, Yves Bertrand, Carine Domenech

**Affiliations:** 1 Institute of Pediatric Hematology and Oncology, Hospices Civils de Lyon, University-Lyon1, Lyon, France; 2 Department of Pediatric Ophtalmology, Hopital Nord Ouest de Villefranche sur Saône, Gleize, France; 3 Department of Pediatric Hematology, Hôpital Robert-Debré, Paris, France; 4 Department of Pediatric Hematology, Hôpital Armand-Trousseau, Paris, France; 5 Department of Pediatric Hematology, CHU Grenoble Alpes, Grenoble, France; 6 Department of Pediatric Hematology, CHRU de Tours, Tours, France; 7 Apoptosis, Cancer and Development Laboratory - Center of Research of Cancerology of Lyon, INSERM U1052-CNRS UMR5286, Lyon, France; 8 Department of Pediatric Hematology, Centre hospitalier Félix-Guyon, Saint Denis de La Réunion, France; 9 Department of Pediatric Hematology, CHU de Limoges, Limoges, France; 10 Department of Pediatric Hematology, CHU de Toulouse, Toulouse, France; 11 Department of Pediatric Hematology, CHU de Saint Etienne, Saint Etienne, France; 12 Department of Pediatric Hematology, CHU de Besançon, Besançon, France; 13 Department of Pediatric Hematology, CHU de Nice, Nice, France; 14 Department of Pediatric Hematology, CHU de Brest, Brest, France; 15 Department of Pediatric Hematology, CHU de Nantes, Nantes, France; 16 Department of Pediatric Hematology, CHU de Lille, Lille, France; 17 Department of Pediatric Hematology, Hôpital de la Timone, Marseille, France; 18 Department of Pediatric Hematology, CHU de Bordeaux, Bordeaux, France; 19 Department of Pediatric Hematology, CHRU de Nancy, Nancy, France

**Keywords:** Childhood, ophthalmic relapse, ALL

## Abstract

Ten to fifteen percent of children with acute lymphoblastic leukemia (ALL) relapse following treatment. Of these, less than 2% display ophthalmic relapses, which owing to their scarcity, are largely undocumented, leaving clinicians with few diagnostic and therapeutic recommendations, despite serious functional sequelae. We conducted a French multicenter retrospective study to collect all clinical, radiological, biological, and therapeutic data, and outcomes for children with ALL ophthalmic relapses. From 2000 to 2020, 20 ophthalmic relapses occurring after first-line therapy performed before January 1^st^, 2017 were included in our study: 14 B-ALL and 6 T-ALL. Fifteen patients (75%) had concomitant involvement of the central nervous system, and 11 (55%) a combined bone marrow relapse. Only 1 had an isolated ophthalmic relapse. Eight children (40%) died, 7 from a refractory disease and 1 from toxic death, and 4 patients relapsed. With a median follow-up of 63.1 months, 8 patients are currently alive in continuous complete remission with only 2 displaying severe ophthalmic sequelae. Although rare, ophthalmic relapse could have a significant impact on the functional prognosis of survivors. Their management must be multidisciplinary, with a central role given to ophthalmologists.

## Introduction

Acute lymphoblastic leukemia (ALL) is the most common pediatric cancer (75-80% of childhood leukemia). Although overall survival at 5 years reaches 85-90%,^1^ 10-15% of children relapse and display a poor prognosis that has not significantly improved over the last two decades.^2^-^6^ The main sites of relapse are the bone marrow (BM) and the central nervous system (CNS). Combined or isolated extramedullary involvements may also be observed in the testis, ovary, eye and kidney.^7^ Ophthalmic relapses, the incidence rate of which is estimated at 2%^8^,^9^, have essentially been reported in case studies on children.^10^-^13^ The clinical signs are in no way specific and may vary according to the part of the eye involved at relapse, as well as the mechanism of blastic invasion (meningeal tropism by the optic nerve or vascular involvement). Leukemic involvement of the retina is frequent^14^ and could be responsible for a progressive or sudden decrease in visual acuity, and in some cases complete loss. Other sites of involvement may include the orbit, cornea, anterior chamber, vitreous, choroid, sclera and may result in painful red eyes and hypopyon.^15^ This non-specific pattern of symptoms may be falsely attributed to a viral infection or a refractive disorder by ophthalmologists, resulting in a delay in diagnostic and therapeutic management, with potential serious functional sequelae.

It is well established that the prognosis of relapses depends on their location and the duration of the first complete remission (CR1).^16^,^17^ Studies suggest that children with ocular relapse, either alone or in combination with a medullary involvement, should be treated with an intensive chemotherapy regimen that includes intrathecal chemotherapy with local ocular radiotherapy of at least 20 Gy.^10^-^12^,^18^ However, data are limited to clinical case descriptions with a short follow-up and no information on the risk of secondary relapse and functional sequelae. Despite the rarity of these leukemic events, their prognosis is severe, and the specific treatment margins (including radiotherapy) are not well defined in relapse protocols. Moreover, little is known about their biological characteristics, hampering the adjustment of preventive treatment. Based on a national pediatric cohort, this study describes the early symptoms indicative of ALL ophthalmic relapse, the treatments received as first-line management and during relapse, and the outcomes of these children. This study thus sets the basis for improving the development of future therapeutic strategies.

## Materials and Methods

### Design and inclusion criteria

We performed a national multicenter retrospective study in the 28 pediatric hematology centers of the “Société Française de lutte contre les Cancers et les Leucémies de l'Enfant et de l'Adolescent” (SFCE). All patients (younger than 20 years) who suffered from an isolated or combined ALL ophthalmic relapse between 2000 and 2020 were included in our study, except patients who received first-line therapy after January the 1^st^, 2017. Patients were treated according to national and European ALL protocols^19^,^20^ in frontline and at relapse. All demographic, clinical, biological and radiological data, therapies and outcomes were collected from the time of ALL diagnosis onwards.

### Definitions

After achieving CR1, ophthalmic relapse was defined as the appearance of clinical symptoms of active ocular disease, associated with an abnormal ophthalmic examination (fundus retinography, optical coherence tomography (OCT) or B-ultrasonography). Clinical symptoms with or without optic nerve infiltration, included red or painful eye, decreased visual acuity, visual field amputation, exophthalmia and diplopia. The eye segment involvement was defined in the **Supplemental file 1**. Cytological confirmation, performed by an anterior/posterior segment puncture was not necessary for diagnosis of ophthalmic relapse if documented by CNS involvement in lumbar puncture. CNS relapse was defined by at least five leukocytes per microliter in cerebrospinal fluid and cytological evaluation, demonstrating the presence of lymphoblasts or clinical evidence of cranial nerve involvement, regardless of the number of cells (status CNS3). Combined relapse was defined by an ophthalmic relapse associated with BM involvement and /or CNS involvement. BM involvement was diagnosed according to the morphological detection of lymphoblasts, and also at the level of the minimal residual disease (MRD) through flow cytometric profiles or using Ig-TCR clone-specific probes defined at diagnosis.

Overall survival was defined as the time from initial ALL diagnosis to death or last follow-up.

### Ethics statement

The patient's legal guardian signed an informed consent before treatment, which included authorization for the use of their data. Data collection was performed in accordance with patient confidentiality. Additionally, parents were informed of this retrospective study by an information note sent by mail or given in person, and did not object. This procedure complied with the Declaration of Helsinki and with the MR004 (“Commission Nationale de l'informatique et des libertés” n°2211365). The SFCE ethics committee approved the study (CL-2020-4).

### Statistical analysis

Descriptive statistics were reported in terms of absolute frequencies and percentages. The distribution of data was described in terms of a median value. Sample odds ratios (OR) were calculated with the R package Epi. P-values < 0.05 were considered to be significant.

Survival analyses included the interval between initial ALL diagnosis and the occurrence of death or the date of last examination, with a five-year follow-up. The cut-off date for these analyses was June the 15^th^, 2021. Survival rates were estimated using the Kaplan-Meier method.

## Results

### Demographic and clinical data of ALL at initial diagnosis

Between 2000 and 2020, a total of 20 patients were included (10 boys and 10 girls) **(Table 1).** Among them, 14 had a B common precursor (BCP)-ALL and 6 had a T-ALL, including one patient with mixed phenotype acute leukemia at diagnosis that evolved into lymphoid at relapse. The overall median age was 6.7 years (range, 0.36 to 15.7 years) at initial diagnosis, with 5.1 years for patients with BCP-ALL and 14.3 years for T-ALL. Five (25%) children had good prognostic features at 1^st^ diagnosis (age, leukocytosis, hyperdiploidy (> 50 chromosomes), ETV6-RUNX1, CNS1 status). Among the 20 patients, 6 displayed a CNS3 status, and 12 had hyperleukocytosis (>50G/l). In the case of (BCP)-ALL (n = 14), 2 patients had a t(1;19) translocation, 2 had an IKZF1 deletion, 1 a KMT2A rearrangement and 1 patient had a Philadelphia chromosome. A summary of the risk factors according to immunophenotype is presented in **Table 1**.

Four patients (20%) were included in the standard risk group (BCP-ALL n = 4; T-ALL = 0), 5 in the average risk group and 11 in the high-risk group according to different French frontline protocols. Ten patients (50%) displayed criteria of poor response to treatment (poor prednisone response at day 8 n = 5; minimal residual disease (MRD) at the end of induction >10^-3^ n = 7, MRD at the end of consolidation >10^-3^ n = 2), including 2 with good prognostic factors at diagnosis. Two patients received immunotherapy with blinatumomab due to a chemo refractory disease in first-line therapy.

Four children underwent allogenic hematopoietic stem cell transplantation (HSCT) before ophthalmic relapse: 3 in CR1, due to their severe and refractory disease, and 1 after initial CNS leukemia relapse. Four children had cranial radiotherapy before ophthalmic relapse: 3 had undergone a 12 Gy total body irradiation (TBI) in the conditioning regimen of HSCT and 1 had received prophylactic cranial irradiation of 12 Gy according to his CNS status (EsPhALL protocol). The 4^th^ patient who underwent HSCT before ophthalmic relapse did not receive TBI as he was an infant.

### Clinical presentation and treatment of ophthalmic relapses (Table 2)

**Figure 1:**
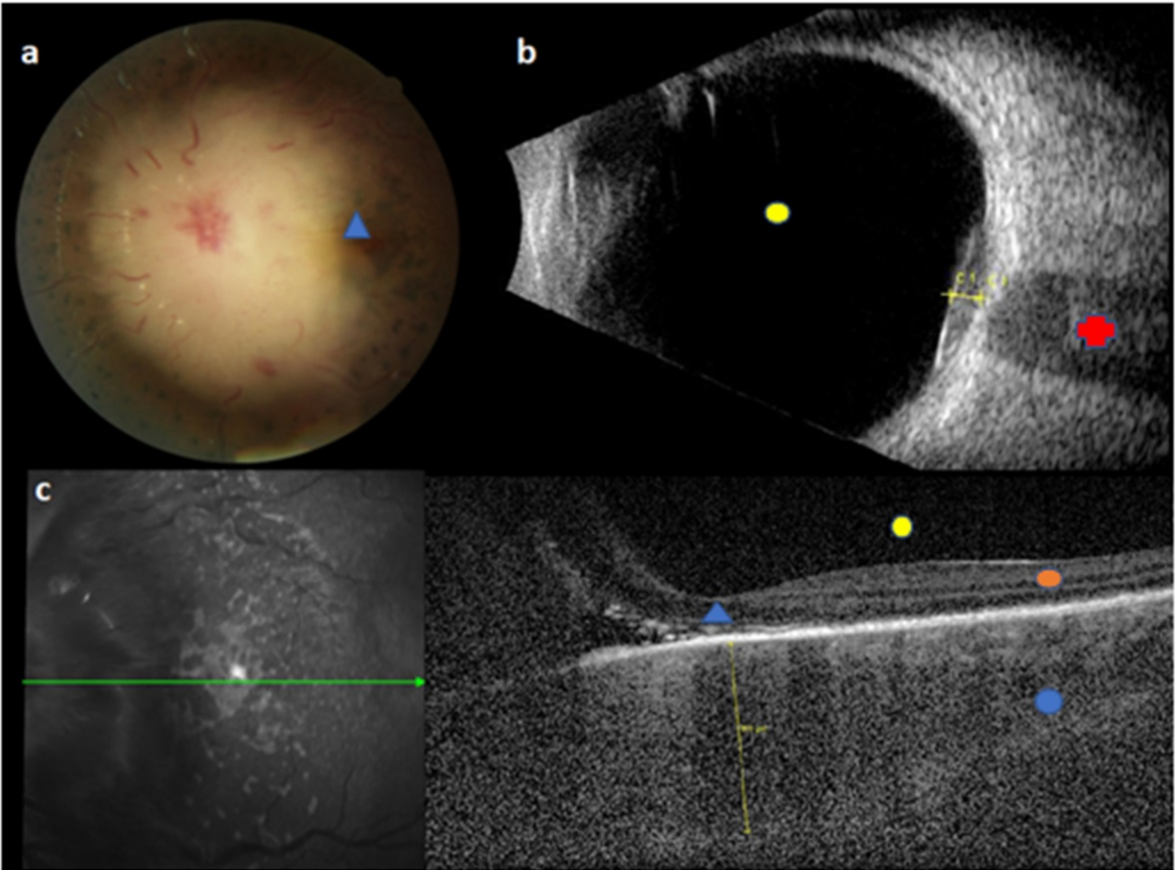
Initial images of BCP-ALL ophthalmic relapse for a child with normal brain imaging and no blast at CSF. This figure reveals a sub-macular choroidal hypertrophy and choroidal infiltration above the optic nerve. **a.** Fundus retinography: choroidal infiltration above optic nerve; all big papillary vessels and papilla are hidden by the choroidal infiltration, blue triangle = macula. **b.** B-ultrasonography: C1 = infiltration above optic nerve; red cross = optic nerve; yellow circle = vitreous cavity. **c.** left picture: fundus retinography; green arrow = horizontal line of OCT. Right picture: horizontal OCT: yellow line = sub-macular choroidal hypertrophy; blue circle = choroid, orange circle = retina; yellow circle = vitreous cavity, blue triangle = macula. Legend: ALL: acute lymphoblastic leukemia; CSF: cerebrospinal fluid; OCT: optical coherence tomography.

**Figure 2:**
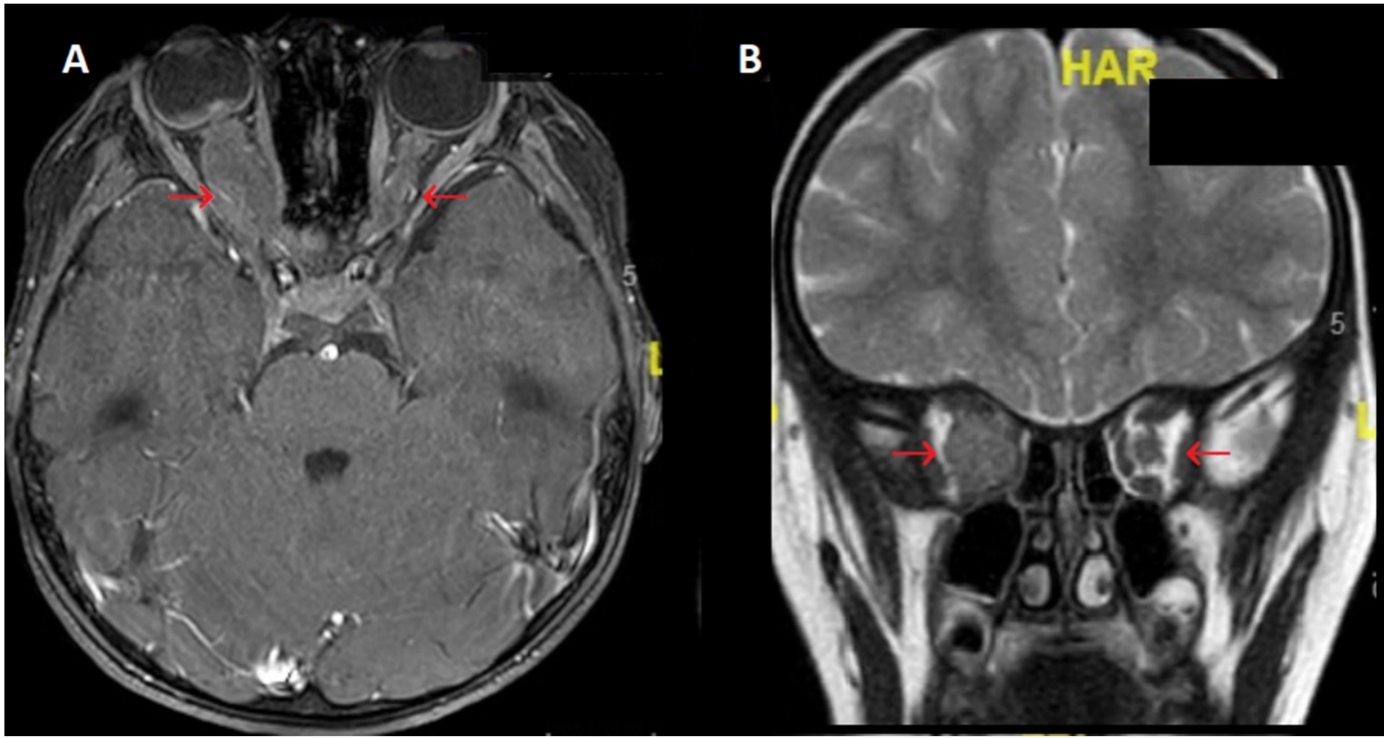
Brain magnetic resonance imaging (MRI) of BCP-ALL ophthalmic relapse for a child with massive infiltration of the right optic nerve. Images show a major circumferential thickening of the homogeneous right optic nerve with little contrast with lesser involvement of the left optic nerve. A. Axial THRIVE sequences after gadolinium injection B. Coronal T2. Legend: ALL: acute lymphoblastic leukemia

Ophthalmic relapse was the 1^st^ relapse for 18 children. In the 2 other patients, ophthalmic relapse occurred after a BM relapse and a CNS relapse. The median age at ophthalmic relapse was 9.9 years (range 1.8 to 18.6 years). Relapse was diagnosed with a median delay of 24.7 months (range, 5.5 to 129.4 months) after the initial ALL diagnosis, occurring earlier in patients with T-ALL (9.9 months *vs* 33.1 months for patients with BCP-ALL; p value = 0.06). Eleven children (55%) relapsed early (< 30 months after initial diagnosis). Characteristics of ophthalmic relapses are described in **Table 2**. The eye segment involvement was anterior for 6 children. Ophthalmic symptoms appeared progressively (more than 15 days) for 8 children, whereas the remaining 12 (60%) had acute symptoms. Patients displayed variable clinical manifestations: the most frequent symptoms being reduced visual acuity (n = 12) and ocular pain (n = 5). Six patients had a bilateral ophthalmic involvement (30%). Nine of the 16 patients with available fundus images (56%) had papilledema related to blastic tumor invasion (**Fig. 1**), 1 (6%) had exudative retinal detachment (sign of choroidal damage). None of our patients displayed signs of leukemic retinopathy, specifically no extensive ocular hemorrhage which is a sign of bone marrow failure. At ophthalmic relapse, 15 children (75%) had a concomitant involvement of the CNS. Among our 20 patients, 18 had undergone brain imaging. The images of 8 of them showed signs of retro orbital invasion: contrast of the optic nerves n = 5; contrast of meningeal n = 1; intracerebral process n = 2 (**Fig. 2**). Among the remaining 10 patients, 5 had an abnormal ocular contrast on cerebral magnetic resonance imaging indicative of leukemia cell invasion, without sign of retro orbital invasion (lens n = 1, retinal n = 1, conjunctival n= 1, orbital wall n = 2) and 5 patients had a normal imaging. Eleven patients (55%) had a combined BM involvement (cytological n = 7; MRD > 10^-2^ n = 2; MRD < 10^-2^ n = 2). Only 1 patient had an isolated ophthalmic relapse. This patient relapsed with an isolated peri-macular lesion 7 months after HSCT, yet displayed a normal cerebral MRI, cerebrospinal fluid (CSF) analysis and negative BM MRD. Despite the lack of documentation on posterior chamber puncture, he was treated with HD-MTX combined with protontherapy before CNS relapse 10 months later. Only 6 patients had cytological confirmation of ocular involvement (4 anterior chamber paracentesis and 2 palpebral biopsy).

Complete treatment of the 20 patients with an ophthalmic relapse is described in **Table 3**. All patients received repeated triple intrathecal therapy (ITT) (hydrocortisone, methotrexate, aracytine) and combined induction therapy including dexamethasone and high dose methotrexate (HD-MTX). However, 2 patients did not receive HD-MTX due to a septic cause or macrophage activation syndrome and myelitis. Half of the cohort underwent allogenic HSCT after remission, including one patient with a second HSCT. Four patients (20%) received autologous chimeric antigen receptor T (CAR-T) cell therapy. A total of 13 children (65%) underwent radiotherapy: 8 underwent TBI of 12 Gy in the conditioning regimen of HSCT, among which 6 patients received an additional cranial boost. Five patients received cranial irradiation without TBI including 1 patient with an 18 Gy protontherapy. Seven children did not receive cranial irradiation including one infant < 2 years of age at ophthalmic relapse and 2 for refractory diseases. Finally, one patient underwent enucleation.

## Outcome

Eight children from our cohort (40%) died: one from toxic death related to HSCT and 7 from refractory diseases including 4 patients who reached medullary remission, albeit with the persistence of an ocular invaded sanctuary site. Among them, 4 patients did not undergo HSCT: 1 because of a late isolated extramedullary relapse and 3 because a new remission could not be achieved. Median time to death after ophthalmic relapse was 13.0 months (range 3.6 to 39.4 months).

**Figure 3:**
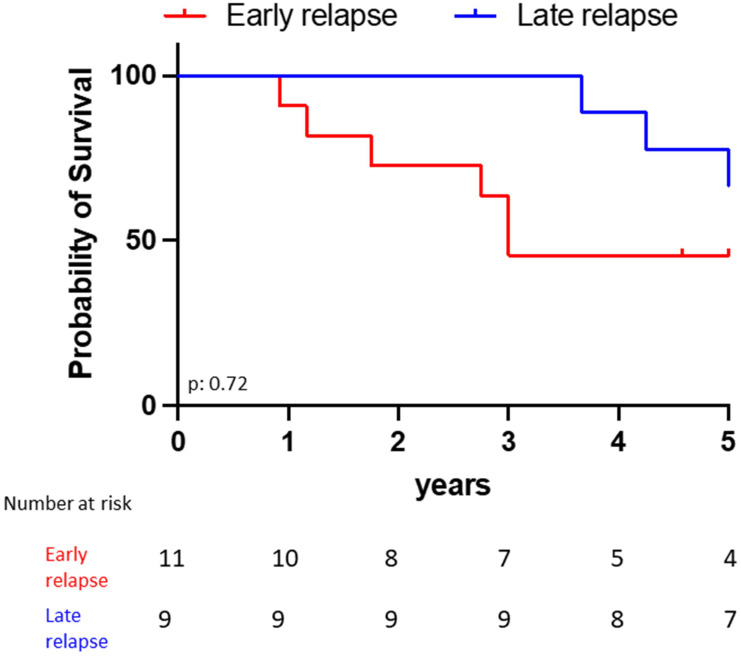
Five years overall survival from initial ALL diagnosis according to the occurrence of early (n=11) *versus* late (n=9) ophthalmic relapse.

The estimated 5-year survival rate after first ALL diagnosis was 63.6% for the late ophthalmic relapses *vs* 55.6% for the early ones (p = 0.72, **Fig. 3**). All patients who received CAR-T cell infusion achieved initial ophthalmic remission. However, 4 patients relapsed when they lost their CAR-T cells between 4 to 6 months after the injection (extra medullary isolated relapse n = 2 (including one periorbital), medullary relapse n = 2).

The 8 surviving children in 2^nd^ complete remission (CR2) (40%) had a median follow-up of 63.1 months after ophthalmic relapse (range 12.3 months to 13.7 years). Two patients had severe ophthalmic sequelae (collapsed unilateral visual acuity with scotoma), and both presented a decrease in visual acuity at relapse. This was unilateral and brutal for one, bilateral and progressive for the other. Importantly, the loss of visual acuity was accompanied by a pale scarred papilla with retinal nerve fiber atrophy.

Characteristics of ALL at initial diagnosis were similar between the patients who died (n = 8), or who are currently experiencing a new relapse or in palliative care (n = 4), and the 8 patients in CR2 (**Table 4**). T-ALL ophthalmic relapses did not have a worse prognosis than BCP-ALL (p = 0.64). Combined medullary (cytological and molecular) relapse was not a significant worse prognostic factor, though it was associated with an odds ratio (OR) of 3.12 (p = 0.36). Furthermore, the use of radiotherapy including TBI (with or without cranial boost) was not associated (p = 0.16) with a better prognosis. Only 2 patients currently in continuous complete remission did not undergo a HSCT associated with TBI after their ophthalmic relapse, but benefited from a cranial irradiation (18 Gy).

## Discussion

Due to therapeutic improvement in pediatric ALL, only 10 to 15% of children will relapse, and of these, only a few will suffer from an ophthalmic relapse. We report here results of an original study on 20 patients, based on the nationwide multicentric inclusion of patients treated in SFCE centers between 2000 and 2020. Most of our knowledge about ophthalmic relapses has so far mainly arisen from case studies in which T-ALL were rarely described, contrasting with our present study in which 30% of ophthalmic relapse occurred with the T phenotype, though this did not constitute a worse prognostic factor than BCP-ALL. This rate was also higher than that reported by Pui et al.^21^

Likewise, clinical manifestations of ophthalmic involvement in children with ALL relapse were described in case reports, but the prognosis of visual acuity was under-investigated.^22^ Even if the pathophysiology is not yet well understood, different mechanisms (more or less related) could explain the variety of ocular symptoms, without specific lesions, observed. Indeed, relapses may have arisen from the CNS, but could also have occurred through the blood, following the rupture of the ocular blood barriers. In the case of “vascular” ophthalmic relapses, clinical manifestations are generally blastic hypopyon, “masquerade syndrome” or choroidal located tumor (diffused hypertrophy and retinal perivascular leukemic infiltrates) that may be associated with a red eye, a painful eye and/or a decrease in visual acuity. In contrast, involvement of the CNS is evidenced by leopard spot retinopathy and/or optic nerve infiltration.^23^ Even though papilledema is well documented in the case of intracranial hypertension, it can also be secondary to a direct optic disc infiltration or due to an optic neuropathy induced by opportunistic infection. Lastly, we also observed ophthalmic relapses in our cohort occurring after immunotherapy, including blinatumomab or HSCT, indicative of a possible mechanism of immune resistance from leukemic cells. We distinguished two different patterns involved in leukemic ophthalmic relapses: leukemic retinopathy *versus* leukemic cell infiltration. Leukemic retinopathy is due to manifestations following BM insufficiency such as retinal hemorrhage or hyperviscosity (roth spots, cotton-wool spots, retinal venous dilatation). Furthermore, ophthalmic relapse is rare and leukemic infiltration is often difficult to diagnose in this context. Hence, the role of ophthalmologists is fundamental, to perform a quick and complete examination, including advanced ocular imaging such as optical coherence tomography (OCT) and fluorescein angiography of retinal vasculature, which may improve early recognition of ocular infiltration.

In view of the seriousness of ophthalmic relapses, it is essential to try to prevent them during first-line treatment. To date, first-line treatment is stratified according to clinical and biological data and on MRD results.^24^-^26^ Based on strategies aimed at minimizing toxicity, the therapeutic program implemented by various groups, such as the EORTC-CLG and the St Jude, includes stopping CNS prophylactic radiation, and has led to some success.^6^,^20^,^27^-^31^ This should be compensated by intensifying systemic chemotherapy with agents that exhibit good CNS penetration and intrathecal therapy. Indeed, prophylaxis of extramedullary leukemia relapses is an important part of first-line treatment. The efficacy of dexamethasone,^20^,^32^ HD-MTX, IT, and Asparaginase for some groups, were demonstrated to prevent CNS relapse, including patients in the low/standard risk groups, and these treatments are part of ongoing European trials.^33^-^35^ Relapse protocols are less well delineated than first-line treatment, and will probably be guided by whole genome sequencing of leukemia cells in future years, with the development of targeted therapies and immunotherapies. Regardless of the site of relapse, patients most often require systemic and intrathecal reinduction chemotherapy. Upon achieving CR2, patients should continue intensive chemotherapy. In addition, some children with poor prognostic factors such as early relapse or high MRD results at the disease evaluation points, may benefit from HSCT after achieving a new CR. The most effective practice is a myeloablative conditioning regimen with TBI, as demonstrated recently.^36^,^37^

In our study, the survival rate of patients is consistent with the overall post-relapse survival rate described in previous studies, including patients with combined medullary relapses.^38^,^39^ The survivors displayed severe functional consequences, including extensive decreased visual acuity to blindness. Visual sequelae are mainly due to retinal, choroidal or optic nerve lesions that can evolve into serious optic atrophy, as well as to toxic therapy (radiation).

Our study proposes new insights into pediatric ALL ophthalmic relapses. Despite their rarity, ophthalmic involvement may be the only early manifestation of leukemic relapse. The diagnosis of any ophthalmic symptomatology concerning a child with a history of ALL should be considered as an ophthalmic relapse until proven otherwise. An urgent ophthalmic consultation (visual acuity, occulomotricity, pupillary defect, slit lamp) is necessary. All oculo-orbital structures should be fully checked by conducting a bilateral fundus examination after pupil dilation, an OCT capable of detecting early choroidal lesions, a B-ultrasonography and a visual field.^40^ Lastly, an angiography (fluorescein or indocyanine green) should be performed to visualize the vascularisation. However, this latter examination is limited due to allergic risks, its invasive nature and the required participation of the child. The ophthalmic consultation must be followed immediately by brain imaging in order to check for retro-orbital damage, especially the contrast of the optic nerves. No corticosteroids (including topical) should be administered prior to diagnostic sampling, including CSF (with cytospin), BM evaluation and anterior chamber paracentesis if needed. The diagnosis of BM relapse currently relies on cytological analysis (blast cells ≥ 5%). However, we recommend that it should be considered as soon as the disease is detectable by flow cytometry and molecular biology (Ig TCR) if at least 1% of lymphoblasts are detected by cytological analysis, as recently proposed by the international consensus of the Pont-di-Legnio Consortium.^41^ Once the diagnosis is confirmed, treatment is a priority. To our knowledge, formal trials have not been performed to evaluate the best treatment options for a rapid clearance of CSF blasts. However, we recommend to use systemic chemotherapies based on good drug penetration into the CNS: DXM (10 mg/m2/day) and HD-MTX associated with repeated triple intra-thecal (steroids, aracytine, MTX) every 4 days until reaching CSF remission. When a HSCT is necessary after obtaining CR, a 12 Gy TBI in conditioning regimen is advised. Due to the small number of patients included in this study, it was difficult to draw conclusions about the therapeutic interest of cranial boost and local treatment by ocular radiotherapy or enucleation. In our study, only one patient underwent enucleation, which did not prevent the progression of the disease. This therapy should thus not be considered as a routine, but may be utilized when the damage is localized in spite of aesthetic sequelae. Indeed, the sequelae would be added to adverse effects of HSCT and TBI, such as radiation retinopathy, cataract or glaucoma. Alternative options that could be envisaged to limit side effects would be protontherapy and contactotherapy. According to the literature, cranial boost does not seem to influence the risk of relapse in children^43^, and concerns exist over potential severe adverse events.^44^ In addition, our study included 3 patients who were treated with Blinatumomab. It would be interesting to complete our findings with further studies to evaluate the efficacy of immunotherapy in these cases of extramedullary relapse. Indeed, even if our 4 patients infused with CAR-T cells experienced a new relapse, Pillai et al reported the regression of a soft-tissue mass involving the eyelid and periorbital region without any therapeutic intervention, consistent with delayed but active tumor cell killing by CAR-T cells.^45^

In conclusion, the management of ophthalmic relapse should be multidisciplinary, with a central role given to ophthalmologists, supported by the use of ocular imaging such as OCT, in the detection of relapse. Future studies should define optimal management approaches for ophthalmic relapses.

## Supplementary Material

Supplementary file.

## Figures and Tables

**Table 1:** ALL initial characteristics of the 20 children with ophthalmic relapse

Patient characteristics	BCP-ALL (n=14) (% of cases)	T-ALL (n=6) (% of cases)	
Male	7 (50)	3	
Female	7 (50)	3	
Age median [min, max]	5.1 y [0.4,12.6]	14.3 y [7.3;15.7]	
< 1 years	1 (7)	0	
1-10 years	10 (71)	2 (33)	
≥ 10 years	3 (21)	4 (67)	
WBC at diagnosis	7 (41)		
< 50 G/L	8 (57)	0	
> 50 G/L	6 (43)	6 (100)	
NCI risk group			
Standard risk	6 (43)	-	
High risk	8 (57)	-	
Status of CNS			
CNS 1	11 (79)	2 (33)	
CNS 2	0	1 (17)	
CNS 3	3 (21)	3 (50)	
Karyotype			
Hyperdiploid	3 (21)	0	
Complex	0	1 (17)	
Molecular rearrangement	1		
t(12;21)/ETV6-RUNX1	2 (14)	0	
t(1;19)/E2A-HLF	2 (14)	0	
t(9.22)/BCR-ABL	1 (7)	0	
KMT2A rearrangement	1 (7)	0	
IKZF deletion	2 /14 (14) evaluated	0	
Treatment group of 1^rst^ line			
Low risk	4 (28)	0	
Average risk	5 (36)	0	
High or very high risk	5 (36)	6 (100)	
Treatment before ophthalmic relapse			
HSCT	3 (21)	1 (17)	
Radiotherapy	3* (21)	1# (17)	
Response (/evaluated cases)			
PPR	1/10 (10)	4	
MRD TP1 > 10-3	6/11 (55)	1/5	
MRD TP2 > 10-3	2/10 (20)	0/5	

Values are the number of patients (%), unless indicated otherwise; Abbreviations: WBC: white blood cell; CNS: central nervous system; HSCT: hematopoietic stem cell transplantation; MRD: minimal residual disease measured after induction therapy (TP1) or after consolidation (TP2); NCI: National Cancer Institute risk group classification. Standard risk: WBC count less than 50,000/μL and age 1 to younger than 10 years. High risk: WBC count 50,000/μL or greater and/or age 10 years or older. PPR: D8 poor prednisone response. *: 2 patients benefited from a 12 Grays total body irradiation (TBI) as a component of HSCT conditioning regimen, and 1 received a craniospinal irradiation due to his CNS3 status (EsPhALL protocol); #: this patient received a 12 Gy TBI before HSCT in 1^rst^ complete remission.

**Table 2:** Characteristics of ALL ophthalmic relapses of 20 children

Characteristics	Patients, n = 20 (%)
Median time from ALL diagnosis [min, max]	24.7 m [5.5, 129.4]
Median Age [min, max]	9.9 y [1.8, 18,6]
Localization of ophthalmological symptoms	
Unilateral	14 (70)
Bilateral	6 (30)
Time to onset of symptoms	
Gradual (>15 days)	8 (40)
Acute (<15 days)	12 (60)
The most frequent initial symptoms	
Decreased visual acuity	12 (60)
Ocular pain	5 (25)
Exophthalmos	2 (10)
Sign of associated meningeal involvement	
Headache	1 (5)
Photophobia	3 (15)
The most frequent ophthalmological examination	
Papilloedema	9/17 (53)
Hypopyon	4 (20)
Combined medullary involvement	11 (55)
Cytological	7 (35)
MRD ≥ 10-2	2 (10)
MRD < 10-2	2 (10)
Combined CNS involvement	15 (75)
CSF involvement	12 (60)
Retroorbital damage at brain imaging	8/18 (44)
Current status	
Complete remission	8 (40)
New relapse or palliative state	4 (20)
Deceased	8 (40)

Values are the number of patients (%), unless indicated otherwise. Abbreviations: MRD: minimal residual disease; CNS: central nervous system; CSF: cerebrospinal fluid

**Table 3:** Characteristics, treatments and final outcome of patients with ophthalmic relapse (n = 20)

		1^st^ line			Previous Relapse	Ophthalmic relapse									
	IP	Blina	RT	HSCT		Relapse delay (m)	HD- MTX (g/m²)	DEX	Triple IT	Blina	CAR-Tcells	TBI/ cranial boost	Isolated CRT (Gy)	HSCT	Status
												(Gy)			
P1	B	-	-	-	-	64.3	1	Yes	Yes	-	-	12/6	-	Yes	CR2
P2	B	-	-	-	-	55.2	1	Yes	Yes	-	-	-	18*	-	CR2
P3	B	-	-	-	-	19.7	1	Yes	Yes	-	-	-	-	-	Died
P4	B	-	-	-	CHT + Bortezomib	74.9	5	Yes	Yes		Yes	-	-	-	Relapse
P5	B	-	-	-	-	13.8	5	Yes	Yes	-	-	12/24	-	Yes	CR2
P6	B	-	-	-	-	34.0	1	Yes	Yes	-	-	-	24**	-	Died
P7	B	Yes	TBI 12Gy	Yes	-	16.1	8 then 5	Yes	Yes	-	Yes	-	Proton 18Gy	-	Relapse
P8	B	-	-	-	-	29.6	5	Yes	Yes	-	-	-	18	-	CR2
P9	B	-	-	-	CHT, TBI, HSCT	58.3	5	Yes	Yes	-	Yes	-	-	-	Relapse
P10	B	-	-	-	-	33.9	5	Yes	Yes	Yes	-	12/6	-	Yes	Died
P11	B	-	-	-	-	53.4	1	Yes	Yes	-		12/12	-	Yes	Died
P12	MPAL	-	-	Yes	-	17.1	3	Yes	Yes	-	Yes	-	-	-	Relapse
P13	B	Yes	-	-	-	5.5	8	Yes	Yes	-	-	12	-	Yes	CR2
P14	B	-	12Gy CS	-	-	32.3	5	Yes	Yes		-	12	-	Yes	Died
P15	T	-	-	-	-	6.9	0#	Yes	Yes	-	-	-	-	Yes	Died
P16	T	-	-	-	-	11.3	0#	Yes	Yes	-	-	-	-	-	Died
P17	T	-	TBI 12Gy	Yes	-	129.4	5	Yes	Yes	-	-	-	-	Yes	CR2
P18	T	-	-	-	-	6.1	5	Yes	Yes	-	-	12/6		Yes	CR2
P19	T	-	-	-	-	12.6	8 then 5	Yes	Yes	-	-	-	24	-	Died
P20	T	-	-	-	-	8.4	1	Yes	Yes	-	-	12/6	-	Yes	CR2

Abbreviations: Blina: Blinatumomab; CAR: chimeric antigen receptor; CHT: Chemotherapy; CR2: second complete remission; ; CRT: cranial irradiation; CS: Craniospinal; DEX: dexamethasone (10mg/m^2^/d); Gy: Gray; HSCT: hematopoietic stem cell transplantation; HD-MTX: High dose Methotrexate; IP: immunophenotype; IT: intrathecal; m: months; MPAL: mixed phenotype acute leukemia; RT: radiotherapy; TBI: total body irradiation including in HSCT conditioning regimen; - : not applicable Relapse delay is delay between the diagnosis and the ophthalmic relapse; # P15 and P16 did not received HD-MTX due to septic cause (P15) or macrophagic activation syndrome and myelitis (P16) *P2 received craniospinal irradiation 18Gy **P6 received cranial irradiation (24 Gy), right ocular irradiation (18 Gy), spinal axis (18Gy) and suffered a 2^nd^ relapse treated with chemotherapy and enucleation, died before HSCT

**Table 4:** Risk (OR) to decease or to have a refractory disease when an ophthalmic relapse occurs (n = 20)

Characteristics	Patients with new relapse or death n = 12* (%)	Patients alive in CR n = 8** (%)	OR	P value
Characteristics of 1^st^ ALL				
Age at ALL diagnosis, median	5.7 y	8.5 y		
[min, max]	[0.36; 14.8]	[2.4;15.7]		
Immunophenotype				
B	9(75)	5(62.5)	1.74	0.64
T	3(25)	3(37.5)		
CNS Status				
CNS 1	7 (58)	6 (75)		
CNS 2	1 (8)	0	2.14	0.64
CNS 3	4 (33)	2 (25)		
HSCT	3 (25)	1 (12.5)		
Radiotherapy	3 (25)	1 (12.5)		
				
Characteristics of ophthalmic relapses				
Time to ophthalmic relapse after ALL, median [min, max]	26.0m [6.9;74.9]	21.7 m [5.5;129.4]		
Unilateral	9 (75)	5 (62.5)	1.74	0.64
Acute appearance symptoms (<15 days)	9 (75)	3 (37.5)	4.56	0.16
Hypopyon	4 (33)	0	-	0.11
CNS involvement	9 (75)	6 (75)	1	1
Medullary involvement	8 (67)	3 (37.5)	3.12	0.36
cytological	6 (50)	1 (12.5)		
molecular	2(17)	2 (25)		
Treatment after ophthalmic relapse				
HSCT	4 (33)	6 (75)	0.18	0.17
TBI + HSCT	3 (25)	5 (63)	0.22	0.17
TBI + Cranial Boost + HSCT	2 (17)	4 (50)	0.22	0.16

*Among the 12 patients: 8 deceased, 4 patients recently relapsed after a RC2 **8 patients = patients among the 12 live patients, excluding the 4 patients in palliative treatment or who recently relapsed. Abbreviations: ALL: acute lymphoblastic leukemia; CAR: chimeric antigen receptor; CNS: central nervous system; HSCT: hematopoietic stem cell transplantation; m: months

## Abbreviations

ALL: acute lymphoblastic leukemia
BCP-ALL: B-cell precursor acute lymphoblastic leukemia
BM: bone marrow
CAR: chimeric antigen receptor
CNS: central nervous system
CSF: cerebrospinal fluid
CR: complete remission
CR1: 1^st^ complete remission
CR2: 2^nd^ complete remission
D: day
DFS: disease-free survival
HD-MTX: high dose methotrexate
HSCT: hematopoietic stem cell transplantation
IT: intrathecal
ITT: triple intrathecal therapy
MRD: minimal residual disease
MRI: magnetic resonance imaging
MTX: methotrexate
OR: odds ratios
M: months
OCT: optical coherence tomography
TBI: total body irradiation
Y: years
