# Supplementary material for: Novel Insights into Pediatric Acute Lymphoblastic Leukemia Ophthalmic Relapses from a Nationwide Cohort Study
Source: J Cancer. 2022 Jan 24;13(4):1272–81. doi: 10.7150/jca.64996 (PMC8899370; doi:10.7150/jca.64996)
Supplement: Supplementary file 1 — Supplementary file. [file jcav13p1272s1.pdf]

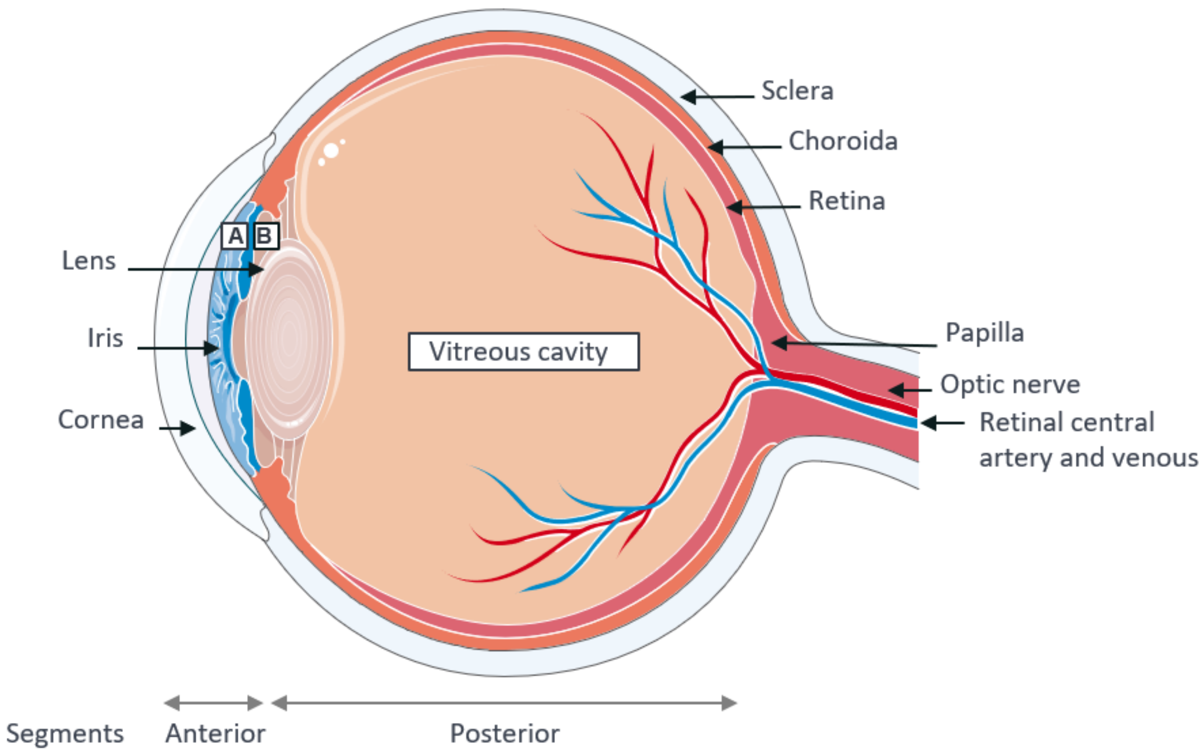

Supplemental file 1: A eye sagittal diagram.

A: anterior chamber; B: posterior chamber.

Anterior and posterior chambers are filled with aqueous humor
